# Application of SERS in the Detection of Fungi, Bacteria and Viruses

**DOI:** 10.3390/nano12203572

**Published:** 2022-10-12

**Authors:** Jiarui Xia, Wenwen Li, Mengtao Sun, Huiting Wang

**Affiliations:** 1 Institute of Health Sciences, Key Laboratory of Precision Diagnosis and Treatment of Gastrointestinal Tumors, China Medical University, Shenyang 110001, China; 2 School of Mathematics and Physics, University of Science and Technology Beijing, Beijing 100083, China; 3 College of Chemistry, Liaoning University, Shenyang 110036, China

**Keywords:** SERS, detection, fungi, bacteria, viruses

## Abstract

In this review, we report the recent advances of SERS in fungi, bacteria, and viruses. Firstly, we briefly introduce the advantage of SERS over fluorescence on virus identification and detection. Secondly, we review the feasibility analysis of Raman/SERS spectrum analysis, identification, and fungal detection on SERS substrates of various nanostructures with a signal amplification mechanism. Thirdly, we focus on SERS spectra for nucleic acid, pathogens for the detection of viruses and bacteria, and furthermore introduce SERS-based microdevices, including SERS-based microfluidic devices, and three-dimensional nanostructured plasmonic substrates.

## 1. Introduction

Surface-enhanced Raman scattering (SERS) is a new technique derived from Raman spectroscopy that can improve sensitivity. It is a kind of enhanced effect spectrum with surface selectivity. When molecules are adsorbed on the surface of the rough metal electrode, the Raman spectral signal of the molecules will be greatly improved, which is called surface-enhanced Raman spectroscopy [1]. In 1974, Fleischman et al. [2] first found high-quality Raman spectra on pyridine molecules on the surface of a rough silver electrode. In 1977, Van Duyne and Creighton et al. [3] presented detailed experimental proof and theoretical calculation of the strong Raman signal obtained from the rough electrode surface and found that the surface enhancement factor of the Raman spectrum was about six orders of magnitude, much higher than the previous thought of tens of times. The huge enhancement effect caused by this rough silver electrode enabled the SERS study. Further advances have been made in the identification of nanostructures, transition metals, single molecule systems, and even biomolecules. Due to the advantages of SERS technology in the detection of biological samples, such as rapid, non-destructive, inhibition of fluorescence background, simple data processing, high sensitivity, and high specificity, it shows a good application prospect in the detection of clinical diseases. With further development, SERS technology is expected to become a new method for clinical disease diagnosis [4].

Judging from the current severe form of COVID-19, a rapid and accurate diagnosis of the virus is very important for preventing and taking effective measures to deal with the disease [5,6,7]. At present, the best methods for diagnosing infectious diseases are: real-time polymerase chain reaction for molecular diagnosis, electrochemiluminescence for immunoassay, and enzyme-linked immunosorbent assay for adsorption assay [8,9,10]. Fluorescence detection technology has now been widely used in the measurement and analysis of viruses and bacteria. Fluorescent labeling agents composed of organic dyes or inorganic quantum dots have been used in bioassays, which are based on fluorescence measurement. Although fluorescence detection technology has good applications, there are still some problems, such as low sensitivity or lack of sensitivity, and this feature is just necessary for the bioassay of specific genes [10,11]. In recent years, some researchers have proposed a new method to solve the problem of fluorescence detection-SERS [12]. When SERS nanoparticles are irradiated with laser, the active site of the incident field will be greatly enhanced due to the local surface plasmon effect [10,13]. However, multiple detections are not a bad aspect, because the width of the Raman scattering peak is much narrower than that of fluorescence emission. Because of these characteristics, SERS detection is more and more widely used in detecting various biological properties [12,14]. For the first time, SERS nanotags are not affected by photobleaching, which is very different from traditional fluorescent dyes [15,16]. At the initial stage of use, SERS technology is used for sensitive immunoassays and can also be used to detect specific markers in tissues or serum [17]. With the development of technology, SERS detection has been extended to the field of gene analysis [18]. According to the latest research, SERS is better than fluorescence-based Raman scattering in terms of analytical sensitivity and multiplexing ability. Today, SERS technology can be used to detect different types of viruses, such as influenza and human immunodeficiency [19], as well as various bacteria [20] Therefore, the research of SERS detection technology in the diagnosis of infectious diseases is of great significance, considering, e.g., the research status, challenges encountered, and wide application.

## 2. Advances of SERS in Fungi, Bacteria and Viruses

### 2.1. SERS Spectroscopy for Fungal Identification and Detection

Fungi are heterotrophic organisms with nuclei and cell walls, and they represent a very large group in the biological world. Generally, they can be divided into yeast, mold, and macrofungi. There are more than 10,000 fungal genera and more than 100,000 fungi in the world. Chitin has obvious characteristics in most cell walls, followed by cellulose. Common fungal organelles include mitochondria, ribosomes, lysosomes, flagella, etc. Common inclusions include crystals, liposomes, etc. In recent years, with the widespread use of broad-spectrum antibiotics, glucocorticoids, immunosuppressants, and various interventional medical technologies, the number and types of clinical fungal infections are increasing year by year, and they have become one of the main causes of nosocomial infections. For example, clinical analysis found that the main source of hospital clinical fungal infections was Candida, among which Candida albicans accounted for about 60% of all infections, and the mortality rate was close to 40% [21] The infection can even kill organ transplant patients, cancer patients, and AIDS patients. Methods and techniques for the efficient identification and monitoring of fungi have attracted much attention.

The traditional fungal detection method is culture and microscopic examination. Culture is the “gold standard” of mycological examination, but it is time-consuming, easy to be contaminated, has low sensitivity, cannot distinguish between colonizing bacteria, and infection bacteria and has other shortcomings [22] Direct microscopic examination is easier and faster, but its accuracy rate is generally not high. It is difficult to provide accurate judgment for early diagnosis of fungi. In recent years, the molecular biology detection method has been research and development hotspot at home and abroad and it has the characteristics of strong specificity and high sensitivity [23] including polymerase chain reaction method, G test method, and GM test, but the cost of this kind of method is high. The operation is complicated and unstandardized, which is easy to produce false positive results in clinical practice. With the development of modern instruments in science and technology, matrix-assisted laser desorption ionization time-of-flight mass spectrometry and loop-mediated isothermal amplification have been applied to the identification and detection of fungi. However, their high requirements for laboratory personnel and expensive experimental instruments have limited their application in rapid clinical diagnosis. Therefore, it is of great research significance and urgent application need to develop and establish rapid and efficient detection methods, technologies, and systems for fungi. Based on the previous research and investigation, we found that Raman spectroscopy, especially surface-enhanced Raman scattering (SERS) spectral analysis technology, has great potential in the field of fungal identification and detection.

#### 2.1.1. Feasibility Analysis of Raman/SERS Spectrum Analysis and Fungal Detection

**Figure 1:**
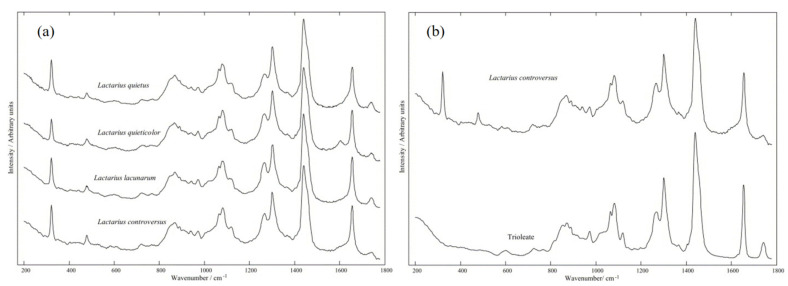
(**a**) Average spectra of amylopectin, amylose, cellulose and chitin polysaccharides; (**b**) Mean spectra of control *Lactobacillus* and trioleic acid. (**a**,**b**) Reproduced with permission of Ref. [25]. Copyright 2004, published by Elsevier B.V.

Raman spectral analysis technology is especially suitable for the detection of biological samples and biochemical systems because of its advantages of providing molecular group structure information, no pretreatment of samples, no moisture interference, and easy to realize real-time detection in situ. As early as 1995, Edwards et al. [24] reported that Raman spectroscopy was used to detect fungal samples from three different genera, and the Raman peaks were assigned. After 10 years, Gussem et al. [25] confirmed that the Raman signals collected were mainly derived from polysaccharides, chitin, and amylopectin on the fungal cell wall and lipids and phospholipids on the cell membrane (Figure 1a). However, because Raman spectroscopy is a spectral analysis technology based on scattered light signals, the scattering signal intensity is weak and the reproducibility is poor, which greatly limits its application in complex living organisms.

In materials science, optics, with the rapid development of instrument science or related fields and the introduction of nanotechnology, the SERS spectra analysis technology has been established, using the rough surface of the metal plasma, in the nanoscale gap within the area (usually 1 nm) to produce hot, greatly enhanced molecular Raman signals of the object under test, which can realize the target of more efficient testing. Based on the continuous in-depth study of SERS spectral analysis theory, it was found that it not only has the basic characteristics of Raman spectral analysis, but also shows strong advantages in detection sensitivity, photobleaching resistance, and facilitates the easy realization of in-situ nondestructive testing, etc. Accordingly, SERS spectral analysis technology has been widely used for the detection of a variety of biochemical samples, such as phospholipid, glucose, glutathione, thrombin, and so on, which has been extended to bacteria and cells and other living organisms in recent years [8], making it possible to detect and diagnose fungi with efficient SERS [26,27,28,29]. Gussem et al. [25] applied Raman spectroscopy to analyze the fungal folds of Lactarius (Figure 1b), analyzed their components, and compared the obtained fungal spectra with Raman spectra of reference substances known to exist in macrofungi, including saccharides. Lipids and minor compounds are used as specific biomarkers (adenine, ergosterol, and glycine). Compared with a large number of literature reports on traditional Raman/SERS detection of small biochemical molecules, there are relatively few reports on SERS detection of fungi as samples. This is because, for fungi, a eukaryotic cell life body, its components are complex and inherently heterogeneous, and its detection characteristics are similar to those of bacteria and cells to a certain extent. It is faced with many difficulties, such as low detection sensitivity, complex signal, poor selectivity and specificity, and poor signal reproducibility and stability. Nevertheless, when breakthroughs have been made in these difficulties in the detection of SERS in fungi, it is reasonable to believe that the detection and related research of SERS for fungi are feasible and have great development potential.

#### 2.1.2. Identification of Fungi Based on SERS Substrates of Various Nanostructures

The SERS enhancement modes mainly include electromagnetic field enhancement and chemical enhancement [30], which are realized by relying on metal, semiconductors, and various composite nanostructures. The structure and efficiency of these active nano basis are particularly important for improving the sensitivity and repeatability of SERS detection. Gold nanoparticles and silver nanoparticles are most commonly applied in SERS detection. By changing the morphology of metal nanoparticles, nanorods, nanoclusters [31], and nanostars [32] were prepared, which can form thermoelectric effect and significantly enhance the Raman signal of the object to be measured. The enhancement efficiency of single metal nanomaterials often fails to meet the requirements of testing, so transition metals, TiO_2_, CuO, ZnO, and other semiconductor materials as well as composite nanomaterials come to people’s attention. At present, a variety of composite nanostructures have shown excellent SERS efficacy, such as putamen nanoparticle structure, two-dimensional Au nanoumbrella structure, polyhedral Ag particles atomic layer deposition nanoparticles, etc. Moreover, metal nanoparticles are combined with a variety of nanomaterials such as magnetic semiconductor materials and organic polymers, and some new structures have better biological compatibility, stronger adsorption capacity, and a stronger enhancement effect, which can achieve more efficient SERS detection. In recent years, with the rapid development of nanomaterials and their preparation technology, the design and preparation of SERS-based nanostructures with orderly and controllable structures have become increasingly mature. Many synthetic preparation techniques and microelectromechanical system processing techniques can be used to prepare SERS substrates with good repeatability, such as the chemical reduction method, magnetron sputtering, electron beam lithography, and self-assembly.

**Figure 2:**
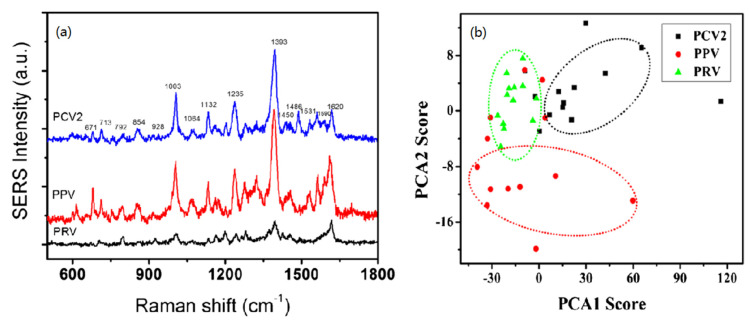
(**a**) SERS spectra of PCV2, PPV and PRV were recorded on the prepared SERS substrate. (**b**) Principal component analysis (PCA) diagram of PC1 and PC2 calculated from SERS spectra of PCV2, PPV and PRV. (**a**,**b**) Reproduced with permission of Ref. [35], Copyright 2017, published by Springer Nature.

**Figure 3:**
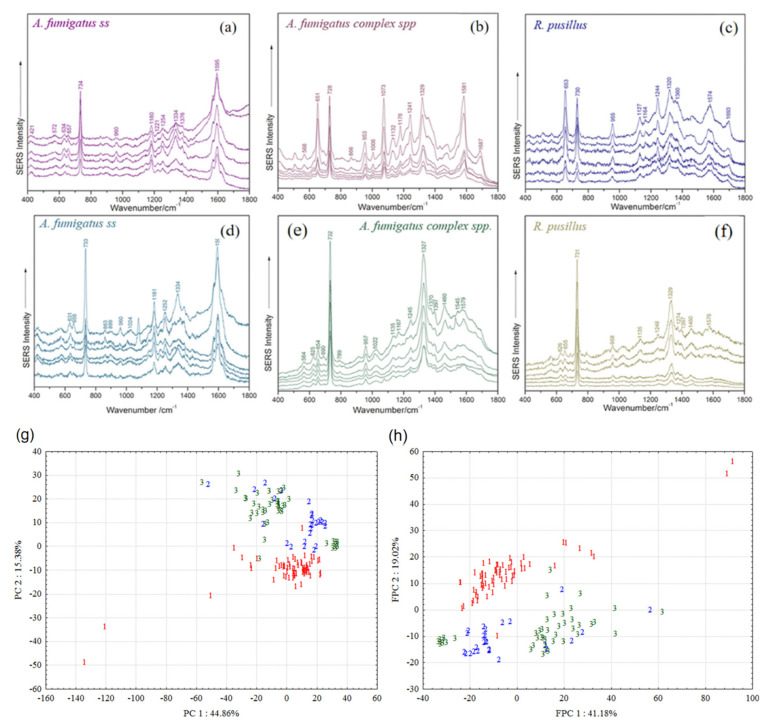
Raw reproducible SERS spectra of fungal samples (*Aspergillus fumigatus*, *Aspergillus fumigatus* complex, Impetigo) recorded using 633 nm laser lines (**a**–**c**) and 532 nm laser lines (**d**–**f**). PC1-PC2 and FPC1-FPC2 have dispersion point plots (**g**,**h**). (**a**–**h**) Reproduced with permission of Ref. [36], Copyright 2018, published by American Chemical Society.

Based on the development and application of various types of nanostructured SERS substrates, in recent years, people began to apply SERS spectroscopy technology to the monitoring and research of viruses, fungi, bacteria, and cells, and their related biochemical processes. Mabbott et al. [33] successfully identified and classified *Candida albicans*, *Candida glabrate*, *Candida cruises*, and *Aspergillus* using silver hydroxylamine nanoparticles modified with single-stranded this DNA as SERS tags combined with principal component analysis. Sivanesan et al. [34] obtained the silver-gold bimetallic composite SERS substrate by electrodeposition of thin gold layer on the rough nano-silver substrate through potentiostatic electrodeposition. After coating with antibiotics, they selectively identified *Escherichia coli*, *Streptococcus enteritidis*, and *Staphylococcus epidermis* from the blood sample. Luo et al. [35] obtained SERS substrate by self-assembling oil-soluble Ag NPs and porous carbon film through hydrophobic interaction. Combined with principal component analysis (PCA), porcine circovirus type 2 (PCV2), porcine parvovirus (PPV), and porcine pseudorabies virus (PRV) were effectively distinguished (As shown in Figure 2a,b). Using nano-Ag as SERS enhanced base, Sivanesan et al. [34] measured the SERS spectra of these three fungi of different genera: Trichophyton, Microsodomia, and tinea epidermis. Using principal component analysis, they realized the effective identification of different subspecies of dermatophytes Trichophyta, which made it possible to identify different subspecies of fungi of the same genus. Dina et al. [36] used the chemical reduction method to synthesize Ag NPs, supplemented by the principal component analysis method and linear discriminant method, and successfully used SERS technology to identify *Aspergillus fumigatus*, *Aspergillus saphenous*, and Rhizopus powder (Figure 3).

The whole process took 5 only min, which greatly shortened the diagnosis time of invasive fungal infection. Prusinkiewicz et al. [37] used the SERS spectrum to detect the inside and outside of fungal cells after co-incubation of Au NPs with *Aspergillus nestulis*, and used the obtained SERS spectrum to analyze the fungal cell environment. Wang et al. [38] established a SERS method for the rapid identification of inactivated *Candida albicans* based on nano-silver glue, and the SERS spectrum after inactivation of *Candida albicans* was significantly different from that before inactivation.

It can be seen that the SERS spectral detection technology integrated with nanotechnology has shown very good development potential and application prospects in the identification of fungal species and the rapid diagnosis of clinical diseases. However, the research and application of SERS in fungal detection are relatively scarce at present, being faced with limitations and deficiencies, such as low detection sensitivity, poor signal stability, and difficulty in signal recognition. The research and development of a more efficient SERS base and nanostructure and the improvement of detection mode can support SERS to play a greater role in fungal detection.

#### 2.1.3. Efficient Quantitative Detection of Fungi Based on SERS Tag Signal Amplification Mechanism

**Figure 4:**
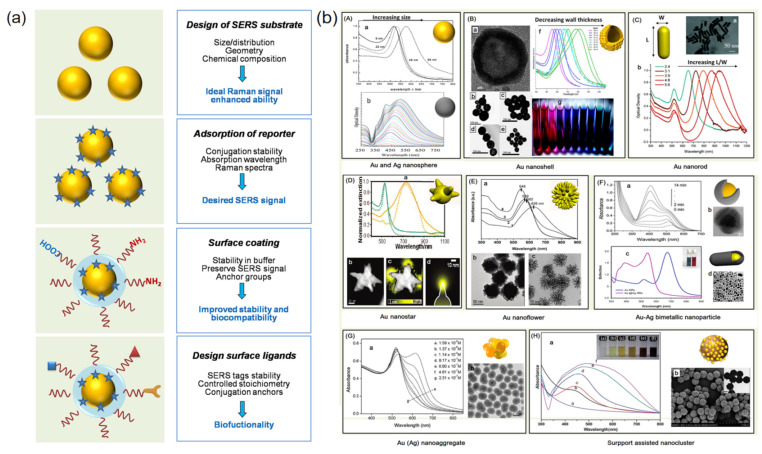
(**a**) Design of SERS tags for biochemical detection. (**b**) Representative nanosubstrates for SERS tags were synthesized. (**a**,**b**) Reproduced with permission of Ref. [39], Copyright 2013, published by American Chemical Society.

When SERS technology is applied to fungal detection, the core problems are sensitivity, reproducibility, and specificity. The characteristics of fungi samples restricted the efficient quantitative test by SERS spectrum. To solve this problem, the SERS tag detection strategy and method were proposed. The design idea of the SERS tag mainly includes the preparation of SERS active metal nanoparticles, the connection of SERS reporter molecules, surface modification, and binding of probe molecules (antibodies, aptamers, etc.), to achieve signal amplification and efficient detection of target components (Figure 4) [39]. Some representative nanosubstrates for synthesized SERS tags are also listed as shown in Figure 4b [39].

SERS tag is composed of metal nano-base material and signal molecules that can provide characteristic SERS fingerprint. Currently, the commonly used signal molecules include 4-mercaptobenzoic acid benzene-4, 4-dithiol, meso-tetraphenyl porphyrin, Ru, rhodamine 6G, malachite green isothiocyanate, etc. [40]. In recent years, a variety of new SERS tags have been designed and prepared, which were successfully applied to the specific detection of biomolecules, pathogens, cells, etc., and even used for the in-vivo imaging of tissues or organs. While improving the sensitivity of SERS detection, they also achieved high selectivity and even specificity detection for specific samples [41].

**Figure 5:**
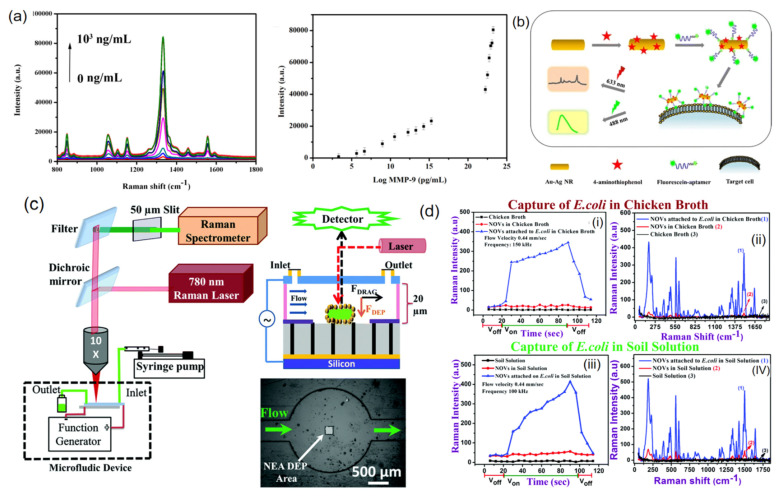
(**a**) SERS spectra of sandwich immune complexes based on AgNPs SERS tags and magnetic microspheres. (**b**) Schematic illustration of the dual mode nanotag preparation and target cell detection. (**c**) Schematic diagram of a microfluidic dielectric electrophoresis device for the detection of bacteria under a Raman microscope. (**d**) DEP capture in *Escherichia coli* cells was assessed by fluorescence and Raman measurements in different complex substrates. (**a**) Reproduced with permission of Ref. [42], Copyright 2019, Elsevier B.V. (**b**) Reproduced with permission of Ref. [43], Copyright 2015, published by Springer Nature. (**c**,**d**) Reproduced with permission of Ref. [44], Copyright 2015, Royal Society of Chemistry.

Zhao et al. [42] prepared a SERS tag consisting of AgNPs and 5, 5-disulfide bis reporter molecules, which was used for immunodetection of matrix metalloproteinase 9CMMP-9 in untreated whole blood samples after conjugation with magnetic nanoparticles through antibody-antigen reaction. The detection limit was 1 pg. M^−1^ (Figure 5a). Bamrungsap et al. [43] assembled the Raman reporter 4-thiophenol and fluorescently labeled aptamer on the surface of gold and silver nanorods by the layer-by-layer assembly method. By synthesizing the dual-function SERS tag, the specific targeting and fluorescence imaging of human protein tyrosine kinase-7CPTK-7 expressed on cervical cancer cells could be realized (Figure 5b). Madiyar et al. [44] used iron oxide gold core-shell nano-ovate particles coated with QSY21 Raman reporter molecules to produce SERS tag, and connected to *Escherichia coli* strain DHα5 through specific immunochemistry to achieve the Raman signal enhancement effect and electrophoretic enrichment effect of SERS tag In combination, the capture time was only 50 s, and the detection limit could reach 210 cfu·mL^−1^ (Figure 5d). Figure 5c shows the schematic diagram of the microfluidic dielectric electrophoresis device for bacterial detection under the Raman microscope. Yuan et al. [45] isolated and detected *Escherichia coli*, *Staphylococcus aureus*, and *Pseudomonas aeruginosa* using antimicrobial peptide-functionalized magnetic nanoparticles as probes for bacterial isolation and gold-plated silver graphene oxide nanocomposites modified with 4-mercapto phenyl boric acid as SERS tags. The lowest detectable concentration for each bacterium was only 10 cfu.mL^−1^. Zhang et al. [46] used vancomycin to modify Fe_3_O_4_@Au magnetic nanoparticles to carry out broad spectrum identification and efficient enrichment of bacteria, and used aptamer functionalized SERS tag to achieve detection limits of 20 and 50 cells.mL^−1^ for *Staphylococcus aureus* and *Escherichia coli*, respectively. Pang et al. [47] used aptamer-Fe_3_O_4_@Au magnetic nanoparticles as magnetic substrates and SERS activated substrates, based on the double SERS enhancement of gold shell and the dual recognition ability of aptamer/vancomycin, for the enrichment and quantitative detection of target bacteria, which could be achieved within 50 mm without the interference of other non-target bacteria. The detection limit of *Staphylococcus aureus* was 3 cells. mL^−1^. These studies showed that the high specificity and sensitivity brought by the SERS tag have great prospects in clinical application.

To further improve the stability of the SERS tag in detection, SERS AG with core-shell nanoparticle structure was developed and developed. He et al. [48] embedded the Raman signaling molecule 3,3-diethylthiotrocarbon-iodine into conjugated gold and silver nanoshells, and the detection sensitivity of methicillin-resistant staphylococcus aureus could be reduced to 300 cfu·mL^−1^. Ye et al. [49] prepared bimetallic double shells by using the seed-mediated method and stepwise nanomachination method, which greatly improved the SERS performance of nanoparticles, and established a new side-flow immunoassay method for surface-enhanced Raman scattering, which promotes the human chorionic membrane. The detection limit of glandular hormone was as low as 0. 077 ng·mL^−1^. Based on the rapid development of SERS tag design and preparation technology, the new SERS tag can further improve the sensitivity and reproducibility of detection while meeting the target analytes for specific detection. The SERS tag has shown effectiveness in the detection of cells and bacteria, and there are also a few studies on fungi. However, the application of the SERS tag in the efficient quantitative detection of fungi will surely attract attention and development.

#### 2.1.4. Efficient Detection of Fungi on Microfluidic Chip Based on Integrated SERS

As is known to all, bacteria, fungi, and cell biological samples usually present basal complex, high background signal, and significantly, the sample is exposed to air and moisture interference under the influence of environmental factors, and the integrated SERS substrate microfluidic chip system and the corresponding test method are used to effectively solve the above problem, showing a clear advantage. Firstly, the Raman laser spot can be directly focused and tested on the channel of the microfluidic chip because of its small size. There are fewer reagents in the microfluidic chip channel which meet the condition of high Raman sensitivity. Because the reaction reagent is not in direct contact, the reaction system does not cause interference. Meanwhile, due to the characteristic fingerprint of the SERS spectrum, the mixture in the reaction system can be analyzed and identified. Therefore, the microfluidic chip integrated with SERS enhanced substrate (abbreviated as microfluidic SERS chip) can provide an efficient testing environment space for biochemical samples and effectively improve the sensitivity, repeatability, and reliability of detection. At the same time, the integration of nanotechnology and microfluidic technology will promote the functional expansion of microfluidic chip, which has broad application prospects in clinical diagnosis, biology, and other fields.

**Figure 6:**
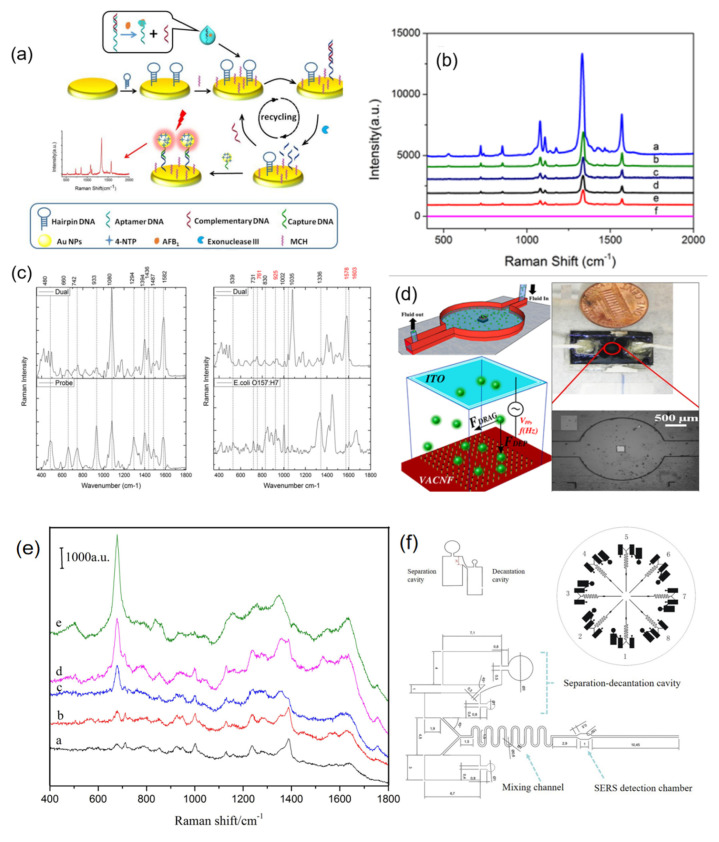
(**a**) Schematic illustration of the SERS sensor for aflatoxin B1 detection. (**b**) SERS spectra obtained in a series of control experiments. (**c**) SERS spectral results of bacterial samples using Nano-DEP microfluidic device. (**d**) Schematic illustration of Nano-DEP microfluidic device. (**e**) SERS spectra of different concentrations of creatinine solution in microfluidic SERS chip. (**f**) Unit structure and size of microfluidic SERS chip. (**a**,**b**) Reproduced with permission of Ref. [50], Copyright 2019, Elsevier B.V. (**c**,**d**) Reproduced with permission of Ref. [52], Copyright 2019, Elsevier B.V. (**e**,**f**) Reproduced with permission of Ref. [29], Copyright 2018, Elsevier B.V.

Li et al. [50] developed Aptamers for a surface-enhanced Raman scattering sensor chip, which can be done in advance, and used the chip function and SERS labels preparation, suitable body complementary DNA hybridization, and other complex operations to successfully realize the mycotoxin detection of aflatoxin BKAFB1), with the linear range of fg·mL^−1^~1 Ng·mL^−1^, and the detection limit can reach 0.4 fg·mL^−1^ (Figure 6a,b). Wang et al. [51] designed a CD-style microfluidic chip to detect fungal PCR products at concentrations as low as 3 ng·mL^−1^ by “printing” an array of oligonucleotide probe lines onto a glass chip using a polydimethylsiloxane polymer plate with radial microfluidic channels, and by hybridizing samples within helical channels. Yang et al. designed an efficient enrichment microfluidic chip for disease spoilers according to the kinetic characteristics of spoiler enrichment at the microscale, and combined with the photoelectric detection system, detected the spoilers of rice smut. Wang et al. [52] realized the specific detection of different subspecies of pathogens at the single-cell level by simultaneously deploying three different SERS probes targeting different epitopes of the same pathogen on the microfluidic SERS chip (Figure 6c,d). The portable SERS chip with a positive charge developed by Yang et al. was successfully used to capture and identify three kinds of urinary system pathogens, including *Escherichia coli* CFT073, *Pseudomonas aeruginosa* PAO1, and *Proteus mirabilis* PRM1, directly from the medium. Su et al. [29] designed a microfluidic chip integrating blood separation, reagent mixing, and SERS detection, and integrated Ag membrane and gold nano SERS base in situ in the detection area (Figure 6e,f). The detection limit for creatinine in water is as low as 4.42 × 10^3^ μmol·mL^−1^, and creatinine in blood samples can be tested within 2 min. Wang et al. [53] adopted the self-assembly and electroless plating composite method to cover the pore surface and inner wall of TiO_2_ nanotubes in the microchannel with Au@Ag nanoparticles as SERS substrate, and the detection limit of R6G was as low as 10^10^ mol·L^−1^. A novel recyclable microfluidic SERS chip was prepared using the photocatalytic properties of Au@Ag/TiO_2_ nanotubes, which could be used as an efficient SERS detector for a variety of biochemical molecules.

A variety of nanostructures in microfluidic chip integration preparation is feasible. SERS spectra collection of reagent and sample is the contact, the biochemical system and the process do not cause interference, and small Raman laser spots are used to focus directly on the channel in a microfluidic chip, used as SERS spectra with sufficient fingerprint performance to get more information. Therefore, we believe that combined with microfluidic technology, our proposed solution is promising. Finally, the current SERS analysis technology has broken through the bottleneck problems, such as low detection efficiency, poor signal stability, and narrow application scope, to provide higher precision and more reliable test means for fungal detection.

### 2.2. SERS Spectra Were Used for Nucleic Acid Identification and Detection

**Figure 7:**
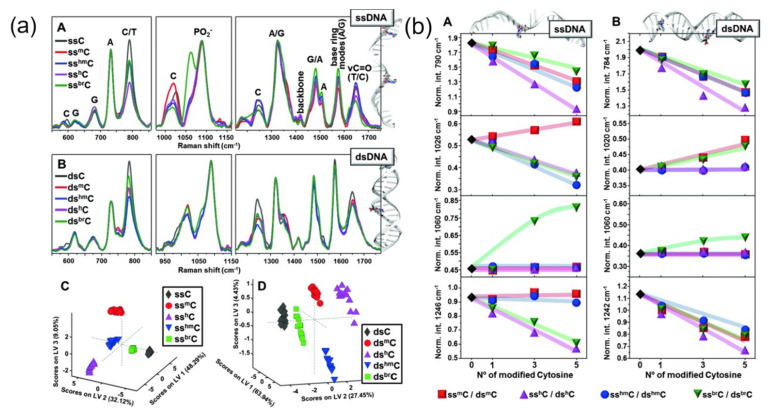
(**a**) Normalized SERS spectra of (**A**) single-stranded and (**B**) double-stranded sequences with different cytosine variations. (**C**,**D**) Partial least squares discriminant analysis of the recorded SERS spectra. (**b**) The relative SERS intensity of C spectral marker band was compared as a function of the content of nuclear base variants. (**a**,**b**) Reproduced with permission of Ref. [56], Copyright 20195, Wiley-VCH Verlag GmbH & Co.

SERS technology has been widely used to study the label free sequence-specific detection of pathogens in biopolymers. The quality and optical properties of plasma substrate determine the accuracy and reliability of data acquisition to a certain extent. Because different surface properties of metal nanoparticles have different effects, researchers have comprehensively studied the important role of the interface of AgNPs in label-free detection [54]. For this reason, researchers have formulated and adopted different preparation schemes for silver colloids to realize the positively and negatively charged surface. Researchers found that there are two mechanisms of interaction between DNA and silver surface. One is mainly due to the displacement of ions or molecules from the metal surface, which is based on the direct adsorption of DNA through its nucleic acid. The other is the electrostatic interaction on the metal surface, acting on negatively charged phosphate skeleton and positively charged additives. The final analysis concludes that the two mechanisms can coexist and their changes will directly affect the contour shape of the SERS spectrum, and the SERS detection technology of DNA nucleic acid modification is reported [55]. The application of positively charged silver nanorods will cause the interaction of phosphate skeleton, thus achieving the aggregation of nanoparticles into clusters in solution. Compared with the SERS experiment, the researchers analyzed four different modifications of cytosine in single and double-stranded DNA samples and recorded the changed SERS spectra [56], which were further analyzed and explained by least square discriminant analysis (see Figure 7a,b).

The analysis of marker patterns in the study of various SERS intensities allows to identify and quantify cytosine variants. The team reported SERS analysis for directly quantifying the nucleobase content of single and double-stranded DNA. The study of this process used cationic nanoparticles as the experimental material [57]. Therefore, under DNA driving, DNA sequences representing different single and double strands are added to the positively charged suspension to aggregate into a relatively stable cluster structure. The relative base content was evaluated by a labeling pattern representing different bases. In addition, the G-quadruplex structure of the human telomere 24-mer sequence stabilized by Na^+^ and K^+^ ions was also evaluated by silver nanoparticles [58]. The author summarized the research on the change of the adsorption mechanism of stable cations on Raman labeling mode. The researchers used silver and gold colloids to conduct SERS research on single and double-stranded DNA molecules [59]. The results show that when ions on the metal surface are easily replaced by functional groups, the adsorption to the metal surface is guaranteed to a certain extent. The author also claims that although the total number of bases is the same, the single sequence on the DNA chain also has a great impact on the SERS spectrum. The latest research expounds on the change in the SERS spectrum caused by the base change in single-stranded DNA, which verifies this conclusion [60]. Researchers confirmed that the contribution of nucleic acid bases added to the DNA strand is the reason for the formation of different SERS spectra. In addition, it was found that the SERS intensity of a specific labeling mode was related to the base position. The closer the base is to both sides, the stronger the SERS intensity is. This result may be due to the existence of the end effect. Finally, we analyzed the species specificity of detection from real samples, such as Phytophthora Ramos [61]. In this study, the whole analysis chain, including the isolation of DNA materials, its polymerase chain reaction amplification, and hybridization with adenine-free capture molecules bound to the metal surface, was considered, and the detection based on SERS technology was also analyzed. Because adenine is replaced in the molecular capture sequence, Raman labeling of adenine can be used to detect the specificity of polymerase chain reaction products. In addition to the label-free test, the researchers also studied the interaction between drugs and double-stranded DNA molecules, which was achieved through the use of electrochemical biosensors [62]. In addition, the interaction between double-stranded DNA molecules and other substances was also studied [63]. In the case of cisplatin, the complexation process was identified in different SERS spectral Raman modes. When methylene blue molecules are added to double stranded DNA molecules, it is found that the spectrum shows a very significant difference from the pure molecular SERS, so it is concluded that the electronic disturbance of molecular structure is caused by the embedding of dye molecules, resulting in a large spectrum difference. It is also found that the relative Raman mode of Hg (II) will shift slightly, and the intensity will be enhanced to a certain extent. Therefore, the analysis is caused by a T-T mismatch. In addition, it is known that aptamers can be used as capture molecules, and the concentration of analyte molecules is related to the conformational change of DNA structure to the metal surface, which can be used to quantify low molecular weight substances [64]. RNA is also used as the second nucleic acid to analyze biological information because of its important functions of encoding and decoding as well as gene regulation and expression [65]. The researchers carried out unmarked SERS technology analysis to obtain specific structural information on RNA molecules. RNA structures with similar surfaces can be classified or identified according to their conceptual information, e.g., example, complementary double-stranded structures or small interfering RNA molecules can be detected, and single base sensitivity can be used to detect quantitative nucleic acid base variants. It is further confirmed that unmarked SERS detection is an ideal candidate for analyzing RNA nucleic acid information. The ultimate purpose of the study is to apply it to the clinical environment. The research results show that the feasibility of label-free SERS detection in clinical application has great potential, which can combine multiple reverse transcriptase polymerase amplification with SERS active substrate (silver nanostructure with a positive charge) [66]. RNA was extracted from urine and the target DNA was amplified into double-stranded DNA. The amplicons were incubated with SERS active nanoparticles and the SERS spectra were recorded. The results showed that different sequences would lead to different SERS spectra. The sources of the differences were attributed to phosphate backbone, purine, and pyrimidine respectively. LCA-PDA was used for biomarker analysis.

In a word, the SERS unlabeled characterization of the interaction of DNA metal nanoparticles shows that the interaction of the skeleton is responsible for the positive surface charge, while the interaction of DNA bases is related to the negative surface charge. A recent direct identification of RNA structure and nucleic acid base variants, which combines the unmarked SERS detection and data statistical analysis, further confirms that the potential advantages of SERS technology in clinical applications have a lot of room to explore, and the portable Raman research system will allow SERS technology to play a role in the clinical environment in the future research process.

### 2.3. SERS Spectra Were Used for Pathogens Identification and Detection

Phytophthora Ramos is a plant pathogen, which can be studied by DNA combined with the specific detection method without markers. In principle, this method is suitable for all pathogens. In addition to these DNA detection-based methods, the whole virus particles are processed through gold nanoparticles, which can be used to detect specific HIV-1 virus-like particles [67]. Researchers detected the characteristic spectral lines at different concentrations, and the results showed that the Raman peaks of carbohydrate, protein, and lipid appeared in the SERS detection results. In addition, SERS technology can also be used to detect bacterial cells. For example, vancomycin can be used to modify the metal SERS active surface to capture bacteria from complex substrates [34].

The SERS spectra of bacteria obtained from blood samples, such as *Escherichia coli*, Enterococcus, and Staphylococcus epidermidis, were recorded. Recently, a research team applied metal-coated polycarbonate membrane to filter cerebrospinal fluid, immobilize pathogens and conduct SERS spectral analysis, which confirmed that clinical cerebrospinal fluid samples can be used to detect and identify meningitis pathogens [68]. The PCA-LDA method was used to analyze and identify related pathogens. In addition, another unmarked SERS method is used to distinguish intestinal streptococcus and *Escherichia coli*, which uses silver dendrites [58]. Here, the method of scanning the surface is used to record the spectrum of bacterial cells adsorbed on the table. At the last stage, *Klebsiella pneumonia* suspension was applied to the SERS substrate and the SERS spectrogram was recorded [69]. Because of the complexity of the SERS spectrum, researchers used multivariate statistical analysis to determine the subtype of *Klebsiella pneumonia*. Silver colloids instead of metal membranes become SERS active surfaces and have been widely used in bacterial detection. *Escherichia coli* cells were incubated with silver nanoparticles and SERS spectrograms were recorded with high reproducibility [70]. The researchers showed that the combination of SERS technology and analysis can successfully identify three strains of *Escherichia coli*. On this basis, a method to detect pathogens of urinary tract infection was introduced. This method is based on electrostatic force to fix bacteria, and the whole process of recording the SERS spectrogram was completed in a very short time [71]. Through principal component analysis, the identification of two different strains of *Escherichia coli* and *Bacillus mirabilis* was achieved. In addition, another research team has proven that the Raman mode sub-signal intensity of the sample combined with silver colloid is much higher than that of the sample synthesized in situ by bacterial cells [72,73]. In a comprehensive study, the author applied the originally prepared silver nanoparticles to the cell wall and studied several bacterial genera [74]. The SERS spectra analyzed and recorded at the single cell level can be used to clarify the distribution of Raman modes of individual components.

**Figure 8:**
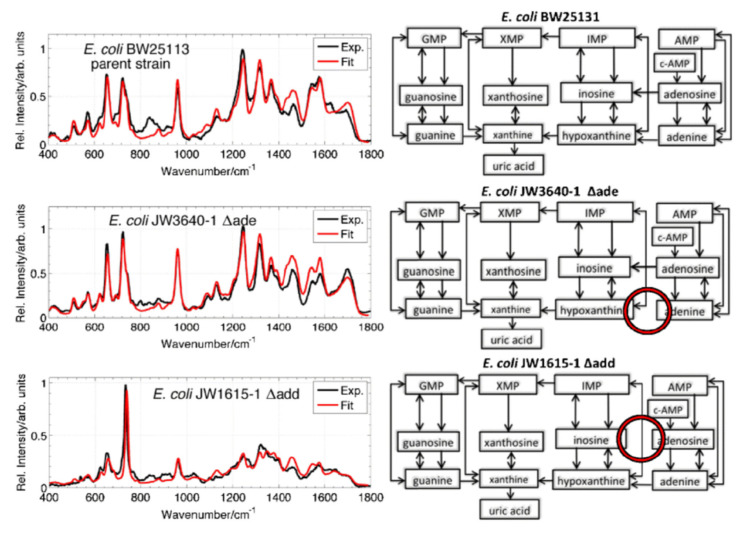
SERS spectra of parent strains and two mutants of *Escherichia coli*, and the corresponding purine metabolic pathway. Reproduced with permission of Ref. [56], Copyright 2015, John Wiley & Sons.

The biological origin of the main cell contribution in the SERS spectrum is clarified in reference [75]. The Raman peak position information of the SERS spectrum is usually determined by the contribution of some adenosine phosphate in the 785 nm excitation wavelength recording table. These components mainly exist on the outer membrane of cells and come from the degradation process, which is caused by bacterial starvation. These results are based on isotope labeling, spectral fitting, SERS investigation, and mass spectrometry analysis. Figure 8 explains that the change in the SERS spectrum is affected by the change in purine metabolism of *Escherichia coli*. The study of this result is to discuss the origin of the bacterial label free SERS spectrum, which provides some support for the subsequent understanding of more molecular-dominated SERS spectra. Recently, the research team published some SERS tests on the identification of 12 bacteria in urinary tract infection samples [76]. Finally, the bacterial cells were transferred to the SERS substrate by centrifugation, and PCA-LDA was used to establish a classification model and successfully identify the urine culture strains. Relevant antibiotic information is also obtained. SERS spectrum of enriched bacteria is controlled by components of nucleotide degradation metabolism.

The particle-based sample preparation scheme is also feasible for enriching bacterial cells from complex matrices. This method has confirmed that the combination of magnetic microspheres and gold silver nanoparticles can be used to achieve sensitive detection from tap water or milk enriched with *Escherichia coli* and *Staphylococcus aureus* [77]. The SERS signal of bacteria differentiation by detection was significantly reduced. To improve the reproducibility of the SERS spectrum, the microfluidic equipment is combined with SERS technology and automatically measured at high flux. Mycobacterium related to pulmonary tuberculosis is destroyed by the module, which uses the laboratory chip SERS for detection and identification [78]. This process does not purify the suspension, so it allows all cell components in the cell to interact with the metal surface. By recording the individual SERS spectra of each sample, the researchers found that the SERS spectra were mainly determined by the contribution of mycotic acid. The researchers used a hierarchical analysis model to identify six different mycobacteria, including the Mycobacterium tuberculosis complex and non-tuberculosis mycobacteria.

Recently, it was found that *Salmonella* and *Cronobacter* were detected in fish, milk powder, and other food substrates [79]. The researchers will prepare the 24-h culture according to the standard sample preparation steps of the International Standards Organization and fix the bacterial suspension on the SERS substrate to record the spectrum. The final result shows that the analysis time will be greatly shortened by using SERS technology for analysis and identification, so SERS technology is undoubtedly a substitute for standard biological diagnosis, and its future application prospect is very broad.

### 2.4. SERS-Based Microdevices for the Detection of Viruses and Bacteria

Microfluidic chip analysis technology can not only integrate several basic operation units, such as sample preprocessing, separation, and detection, but also combine with a variety of detection technologies to achieve high-throughput and high-connotation detection of biochemical samples. It has shown outstanding advantages and potential in the research of bacteria, fungi, and cells. By integrating the SERS analysis module on a microfluidic chip, biochemical samples containing fungi can be introduced into the set integrated nanostructured SERS detection micro area of the microfluidic chip, and the in-situ detection and biochemical information can be obtained directly, which provides a new opportunity and a new way for efficient detection of the biochemical system. At present, many types of infectious disease researchers have tried to use SERS for research and have made many infectious disease micro devices based on SERS [80]. The following is a summary of SERS devices in recent years. Microdevices based on SERS have received great attention from many industries, such as materials science, analytical chemistry, medical analysis, and diagnosis [81,82,83]. Therefore, it is very important to update and develop the development of micro devices in time. What kind of breakthroughs will the related technologies bring, and how to accelerate the development of the analysis platform based on SERS technology, will improve the speed and accuracy of disease diagnosis to a certain extent and have important applications in the diversification of application fields. What is particularly important is that, recently, microdevices based on SERS technology have shown wide applicability in detecting viruses.

This section mainly describes three different SERS microdevices. In recent years, researchers have focused on how to improve the reproducibility of biosensor devices because of some difficulties in SERS detection, e.g., the SERS active site is difficult to replicate, and the bioanalytical target must be quantitatively analyzed. Due to the different types of microdevices, the new SERS detection has some improvements in controlling reaction time, local heating, or light separation. These devices can achieve the repeatability and quantitative results of SERS technology to a certain extent. In recent years, micro devices developed for biomedicine, such as microfluidic chips and nanostructured plasma substrates, have provided many conveniences for medical work, so micro devices based on SERS are of great significance for biomedical diagnosis. Similarly, we should be aware that the close relationship between the medical and scientific communities is very important for creating better detection technologies, so we still need to work hard to consider the current challenges faced by microdevices in virus and bacterial diagnosis.

#### 2.4.1. SERS-Based Microfluidic Devices

In the early diagnosis of infectious diseases, rapid and sensitive detection technology is crucial in the clinical detection of pathogens and viruses, so it is of great significance to develop a new detection technology. Recently, microfluidic devices based on SERS technology have attracted much attention due to their great application potential in diagnosis. The application of SERS in repeatable POC diagnosis has attracted many researchers’ attention. As SERS technology is faced with the problem of enhanced reproduction in quantitative analysis of biological targets, the root of this problem is caused by factors such as uneven distribution of target molecules. The combination of SERS and microfluidic technology brings the advantages of the traditional macro environment and new ideas to solve problems. For example, the homogeneous mixing conditions generated in microfluidic channels have been proven to be helpful for SERS quantitative analysis. In addition, the combination of the two also realizes the ideal mechanism environment of high sensitivity and repeatability. The other advantages of this integrated system are low consumption and high detection speed.

**Figure 9:**
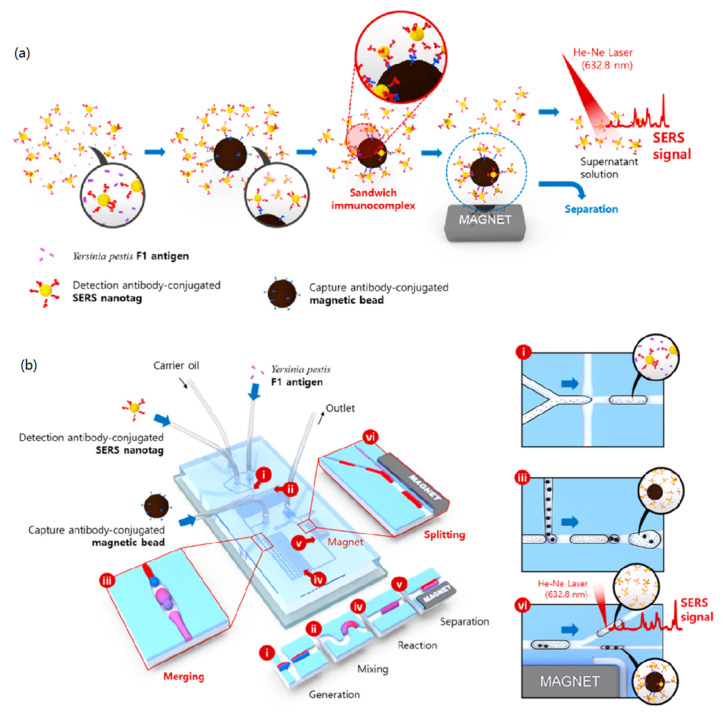
(**a**) Schematic diagram of F_1_ antigen detection immunoassay platform based on SERS nano tag and magnetic beads. (**b**) Schematic design of the integrated SERS-based microfluidic channel composed of six microdroplet compartments. (**a**,**b**) Reproduced with permission of Ref. [84], Copyright 2017, American Chemical Society.

SERS has the characteristics of high sensitivity, while the characteristics of microfluidic devices can reduce the volume of samples, and the ability to automatically process analytical samples is the basis for better integration of the two. Therefore, the advantages of both make the instrument formed by SERS microfluidic detection equipment have a good application prospect in the detection of viruses and bacteria. Choi et al. [84] studied a SERS microfluidic device for automatic immunoassay (1) of partial antigen 1 in *Yersinia pestis* (Figure 9a). Due to its potential use in biological weapons, the device has aroused the interest of national defense institutions and it has been proposed to modify the instrument for multiple micro drop operations, including micro drop generation, promoting rapid and automatic immune response, etc. This platform is considered to be a multifunctional SERS microfluidic platform. Through this platform, multi-step analysis can be carried out, and harmful pathogenic bacteria can be detected more accurately and safely.

**Figure 10:**
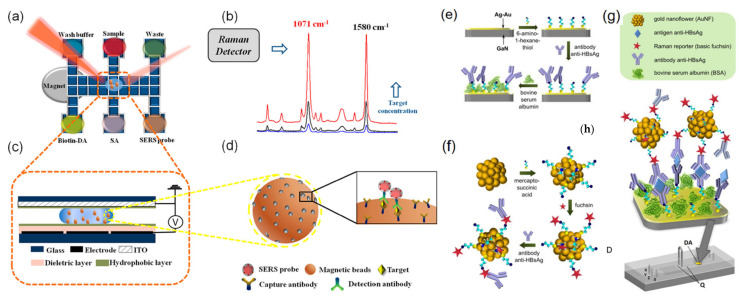
Schematic illustration of SERS-based immunoassay with digital microfluidics. (**a**) Illustration of DMF-SERS method and bottom plate of DMF chip. (**b**) Two characteristic Raman peaks of 4-MBA at 1071 and 1580 cm^−1^. (**c**) Side view of DMF chip containing a droplet with magnetic beads. (**d**) Immunocomplex functionalized with SERS tags on magnetic beads. (**e**) Sequential steps for the formation of the SERS-based. (**e**) The capturing substrate preparation, (**f**) the Raman reporter-labeled immuno-Au nanoflowers synthesis, and (**g**) SERS detection of the sandwich interactions, (**h**) schematic illustration showing the integration of a microfluidic device with the SERS-active substrate based on Au–Ag coated GaN surface. DA, detection area chamber with GaN/Au–Ag SERS substrate. (**a**–**d**) Reproduced with permission of Ref. [85], Copyright 2018, American Chemical Society. (**e**–**h**) Reproduced with permission of Ref. [86], Copyright 2014, Elsevier B.V.

Wang et al. [85] developed a device for quantitative analysis of avian influenza virus in human serum, which is a SERS-based digital microfluidic (DMF) automatic device, as shown in Figure 10a. When the equipment is working, it only needs to apply a simple composition to the electrode array to achieve the effect of driving the drop independently, so as to realize the original transportation and even distribution process. For the sandwich immunoassay method designed, the magnetic bead coated with antibody is selected as the solid carrier, so as to achieve the effect of capturing the antigen from the sample to form the magnetic bead antibody-antigen immune complex. This method can greatly improve the SERS signal and sensitivity. At the same time, the automation function of DMF reduces the risk index of exposure to dangerous samples. The most noteworthy work can complete the detection of avian influenza virus in human serum within 1 h. Kaminńska et al. [86] also used this platform to test hepatitis B virus antigen in human serum and made some achievements (Figure 10e–h).

In the above equipment and devices, it is clear that target reagents of various concentrations should be prepared before quantitative analysis, because it is a time-consuming process to repeat the liquid transfer process first, and there will be experimental errors for target reagents of different concentrations, which is a problem that must be solved. In order to overcome this problem, Jeon et al. [87] developed a programmable automatic gradient device, which can directly generate target reagents of various concentrations. As shown in Figure 10a–d, the gradient module is embedded in the original system, and the target can be detected repeatedly and quickly in multiple concentrations under this module. Compared with other equipment, the system has many advantages, such as easy automation and reduced sample requirements. In a word, the equipment opens a new way to solve the above problems.

#### 2.4.2. SERS-Based Three-Dimensional Nanostructured Plasmonic Substrates

**Figure 11:**
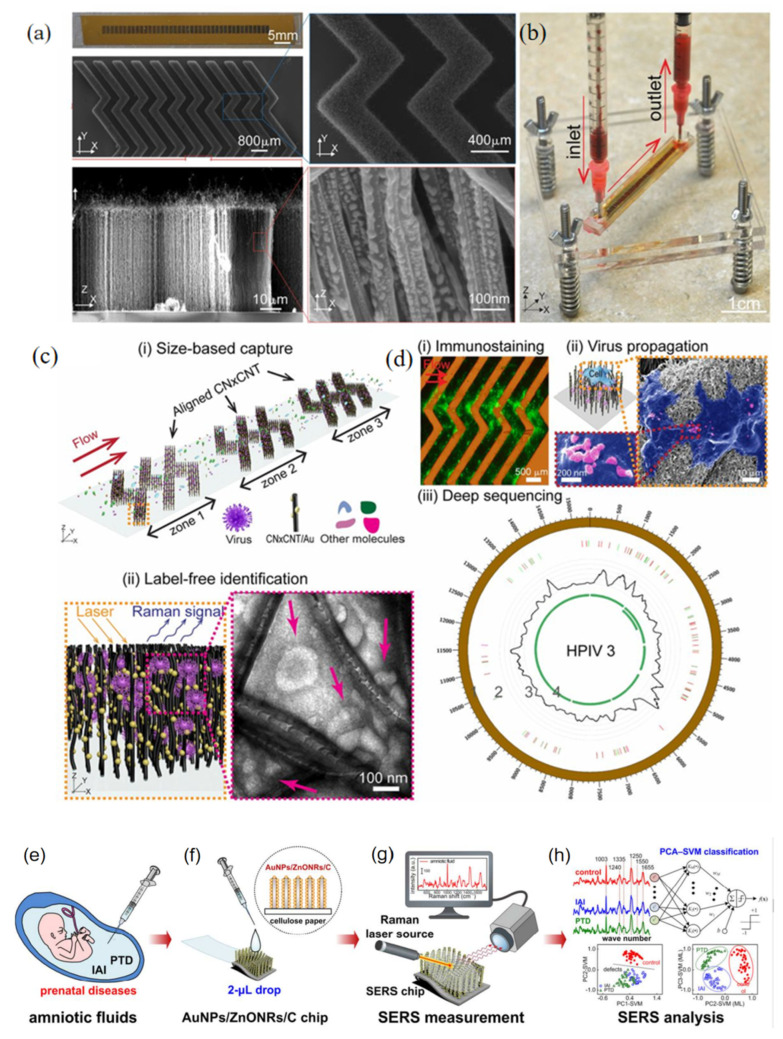
The design and workings of VIRRION for effective virus capture and recognition. (**a**) Photographs and SEM images of aligned carbon nanotubes decorated with gold nanoparticles in A herringbone pattern. (**b**) Images of blood samples being processed by the assembled VIRRION device. (**c**) illustrate (**i**) size-based capture and (**ii**) in situ Raman spectroscopy for label-free optical virus recognition. Electron microscope image of H5N2 avian influenza virus captured using CNxCNT array. (**d**) on-chip virus analysis and enrichment of NGS, (**i**) on-chip immunostaining of captured H5N2, (**ii**) on-chip virus transmission by cell culture, and (**iii**) genome sequencing and analysis of human parainfluenza virus type 3 (HPIV 3). AuNPs/ZnONRs/C chip for SERS analysis of amniotic fluids to detect prenatal diseases. (**e**) Collection of amniotic fluids. (**f**) Dropping 2 μL of amniotic fluid onto the AuNPs/ZnONRs/C chip. (**g**) Real-time Raman measurement. (**h**) Multivariate statistics-derived machine-learning-trained bioclassification method. (**a**–**d**) Reproduced with permission of Ref. [49], Copyright 2019, National Academy of Science. (**e**–**h**) Reproduced with permission of Ref. [88], Copyright 2018, American Chemical Society.

Yeh et al. [49] recently studied a rapid SERS detection platform based on unmarked carbon nanotubes. It is used in the clinic to capture and identify viruses. Researchers have developed a new sample preparation platform, named “Virus capture using rapid Raman spectroscopy detection and identification (VIRRION)”. The platform can capture the target according to the size of the virus and combine the machine algorithm to capture the virus. This method is not destructive, and has more obvious advantages than the previous virus detection methods, provides new inspiration for the subsequent clinical samples’ immune staining, sequencing, etc. Figure 11a–d is the schematic diagram of the platform to identify and capture viruses.

Kim et al. [88] developed a SERS sensor chip, which is a three-dimensional plasma nanostructure with ZnO nanorod arrays grown from cellulose paper and decorated with gold nanoparticles. In this study, it is confirmed that this platform can enhance the Raman signal to the order of 10^7^ and show good reproducibility, which proves that this platform can be used for the detection of various viruses and bacteria and has very good application value for early infectious disease diagnosis. In addition, the platform can also be used for amniotic fluid of patients with infection during pregnancy in combination with machine computing multivariate analysis.

In order to find a SERS substrate more suitable for sensitivity and repeatability determination, Wang et al. [89] made a three-dimensional (3D) gold nanopillar substrate using thermal evaporation. Three mycotoxins can be immunoassay with this three-dimensional substrate and antibody labeling. Because the nano column of the 3D substrate is highly uniform and the peak intensity of Raman scanning is relatively small, Raman spectroscopy is conducive to the stable and repeated analysis and determination of mycotoxins. In a word, the three-dimensional structure substrate can accurately and repeatedly detect the target biological target substances in the Raman scanning area. It can be seen that different SERS substrates are used to detect bacterial viruses, such as silver colloid, silver nanorod, etc. The SERS analysis results of bacteria show that the Raman peak in the SERS spectrum of bacteria is usually related to the degradation process of bacteria. The combination of SERS and micro devices opens up a new idea for automatic detection.

## 3. Conclusions and Prospects

Given the demand for efficient detection of fungi caused by fungal infection in recent years, SERS spectral analysis has shown good research significance and the application potential in efficient detection and identification of fungi with its advantages of rich information, no standard, non-destructive, and in situ detection. Fungus, as a complex organism, involves many substances, as well as much energy and signal conversion in its SERS detection, and its Raman/SERS effect is relatively complex. There are still some problems and challenges in this field. Improving the detection sensitivity, reproducibility, and selectivity is the key to achieving effective identification and testing. By designing and preparing SERS activity-enhancing substrates with controllable structure and good enhancement effect, we develop SERS tags that specifically identify fungi. While improving the detection sensitivity and selectivity, we use the advantages of microfluidic chips to improve the SERS detection environment and enhance the reproducibility and stability of detection. Hence, it is expected to realize rapid identification and detection of fungi in complex samples. Although some achievements have been made in the study of SERS technology in the detection of viruses and fungi and bacteria, the application of SERS technology has not exceeded the traditional laboratory-based sequencing and immunoassay technology. However, SERS showed weak detection ability for complex sample systems, and it was impossible to accurately predict molecular structure information simply by relying on the SERS atlas. Therefore, SERS technology should be combined with other technical means to enhance the detection and characterization ability of SERS and realize the detection of the measured molecules with high selectivity and sensitivity. In addition, SERS is faced with a big challenge, namely the SERS efficiency of nanoparticles used as optical platforms is low, which leads to a long acquisition time. Therefore, how to better improve the stability of the SERS base and how to improve the repeatability of SERS detection are the problems we need to solve. However, we still believe that SERS technology has a good prospect in detection.
